# Evaluating the effectiveness of web-based oral health education on enhancing mothers’ awareness: a semi-experimental internet-based intervention

**DOI:** 10.1186/s12903-024-05070-3

**Published:** 2024-10-26

**Authors:** Kasra Kashani, Arash Shahravan, Afshin Sarafinejad

**Affiliations:** 1 Student Research Committee, Kerman University of Medical Sciences, Kerman, Iran; 2 Faculty of Paramedicine, Department of Health Information Technology, Urmia University of Medical Sciences, Urmia, Iran; 3 Department of Endodontics, School of Dentistry, Kerman University of Medical Sciences, Kerman, Iran; 4 Clinical Informatics Research and Development Lab, Clinical Research Development Unit, Shafa Hospital, Kerman University of Medical Sciences, Kerman, Iran

**Keywords:** Oral health, Child, Dental care for children, Tooth, Internet-based intervention

## Abstract

**Objectives:**

This study examined the impact of web-based education on enhancing mothers’ awareness of oral health care for children aged 9 and 12 years. It focused on the crucial role of mothers in educating about oral health to prevent dental diseases and reduce long-term healthcare costs.

**Methods:**

Mothers were divided into three groups: control, intervention with a web system, and intervention with a brochure. Over one month, their knowledge about their children’s oral health was assessed before and after the intervention.

**Results:**

Data were analyzed using parametric t-tests and non-parametric Mann-Whitney U tests. The findings indicated a significant increase in parental awareness in both intervention groups.

**Conclusion:**

It is recommended that parent education be done using web-based systems or mobile apps, which are better choices due to greater accessibility and interactivity.

## Introduction

### Overview

Oral and dental health, as an integral part of overall human well-being, holds great significance [1]. Oral and dental diseases are among the most prevalent conditions worldwide [2], with considerable social and economic impacts [3]. These problems affect over 600 million children globally [4]. However, policies related to the prevention of these diseases are often overlooked [5]. By instilling hygienic behaviors at a young age, many diseases and complications in later years can be prevented [6]. Children are a vulnerable population with limited information available about their oral health [7]. According to statistics reported in the population of nine-year-olds, the average number of decayed teeth was 0.88, with some regions reporting up to 1.4 [8].

The age of 12 years is specially emphasized by the WHO as the index age for oral health policymaking in adolescents and has been chosen as the key indicator for analyzing global trends in dental decay [9]. One reason for this is the eruption of all permanent teeth by this age [10].

The consequences of neglecting prevention are visible in many countries. For example, the prevalence of Early Childhood Caries (ECC), defined as the presence of decayed, missing, or filled tooth surfaces in children’s primary teeth [11], exceeds 90% in Saudi Arabia and has remained unchanged for over 20 years [12, 13], while in Iranian preschool children aged 5 to 6 years, the prevalence of ECC is over 87% [14]. Education, by raising awareness, can improve access to services and increase the demand for preventive healthcare [15, 16].

### Importance

Children spend most of their day with their mothers, making it crucial to provide mothers with accurate information to increase awareness of children’s dental health [12]. Based on past studies, mothers have a significant impact on their children’s oral hygiene behaviors, highlighting their role in preventing dental decay and oral diseases in their children [15, 17]. Mothers’ knowledge about oral health correlates with behaviors related to their children’s oral health [18, 19]. Mothers have a complete responsibility for monitoring their children’s oral hygiene behaviors during their developmental years, and mother’s awareness in this regard is vital [20].

Traditional education methods using oral instruction and printed materials or videos often cannot meet the diverse learning needs [21]. Technology innovations facilitate presenting relevant and tailored health information [22]. Today, internet-based education has become one of the most appropriate methods of instruction, transforming people’s lifestyles and work, and reducing the time spent on activities [23]. Internet-based education offers numerous advantages, allowing learners to adjust the content, timing, location, and pace of learning according to their needs [21]. Online resources provide quick and easy access to information for medical education purposes and enhance learning experiences [24]. These approaches often focus on achieving behavioral change through dental health education and awareness programs [25].

### Solution

Health education involves the exchange of information with the goal of increasing awareness and knowledge about how to maintain health and prevent diseases, including information about available resources and the benefits of accessing services [26]. Many mothers obtain information about childcare from the internet, and it is suggested that modern technologies be used to deliver education to mothers [21, 27]. Therefore, launching quality websites about childcare is essential [21]. Based on study results, it is recommended that web-based remote education should typically be utilized in educational programs for mothers [27]. The idea of e-services is one of the prominent applications of information and communication technologies in various fields [23]. These findings indicate that this type of web-based remote education can be effective and acceptable [28]. Currently, there are many websites containing medical information about children’s health issues. However, verifying whether these websites provide accurate, qualified, and current information is challenging [21]. The existence of a system or website that provides content alone is not sufficient [21]; today, there is a variety of websites with differences in design, content, and health system management, leading to variations in outcomes such as health and responsiveness [29].

In many of these studies, mothers’ acceptance of web-based interventions has been reported, and many have found the content useful [30]. These programs are expected to be time-efficient and encourage mothers to actively participate in the learning process [27]. In this pathway, by designing an educational information system with interactive capabilities for teaching mothers and children, using appropriate web platforms, a group of mothers will be assisted in learning behaviors related to oral and dental health for their children, and the effectiveness of this tool will be scientifically assessed. It is hoped that the results of this study will assist educational managers and policymakers in provinces in making decisions and policies to enhance students’ oral health literacy [31].

This study aimed to assess the impact of a web-based educational intervention and educational brochures on improving mothers’ awareness of oral health care for their children aged 9 and 12 years in the city of Kerman. The primary focus was on evaluating the effectiveness of two-way educational interactions provided through a web-based system at https://ycoh.kmu.ac.ir and brochures in enhancing mothers’ knowledge and practices regarding oral health.

## Methods

### Methodology

The present study is a semi-experimental internet-based intervention conducted from October 2022 to March 2023. Ethical approval was obtained from the Kerman University of Medical Sciences ethics board under the number IR.KMU.REC.1401.512. Informed consent was collected manually and electronically at the end of the questionnaire from all participants.

### Sampling and selection

The sample consisted of mothers with children aged 9 and 12 years, who were studying in selected schools within the city. The required sample size for a significance level of 0.05 with 95% confidence was 210 participants. However, there was no maximum limit set for the sample size. Based on these considerations, 7 schools with easy access, consent of the school authorities, and homogeneity within the city of Kerman were selected to participate in the study, totaling 495 participants.

### Development of the system and brochures

Initially, the research team categorized and prepared the educational content based on the opinions of pediatric dental specialists and credible sources in the form of videos and texts. Brochures containing general information about oral and dental health based on specialist knowledge and reputable sources were prepared. Additionally, a dedicated system was established with a domain provided by the Kerman University of Medical Sciences, offering extensive content along with features such as interaction with specialists and a Q&A facility (https://ycoh.kmu.ac.ir).

### Sample placement

Participants were randomly divided into three groups based on a random number table: Group A included mothers who had access to the educational web-based system, Group B consisted of mothers who received brochures, and Group C comprised mothers who received neither. Informed consent was collected on the first page of the questionnaires and also in their online versions.

We acknowledge the ethical concerns regarding the control group not receiving educational content during the study. To address this, we ensured that all participants, including those in the control group, were given access to the educational materials after the study concluded. This approach allows us to fulfill our ethical obligation to provide valuable information to all participants while maintaining the integrity of the research.

### Data collection

To assess parents’ awareness of their children’s oral health, a valid questionnaire published before in Ref# [32] was used which included information on privacy, demographic data, and dental care information. This questionnaire, adapted from a similar study, underwent validation through a thorough review by several dental professors, who confirmed its validity through their expert evaluation of the questions and their alignment with the research objectives. Additionally, the reliability of the questionnaire was established with a Cronbach’s alpha of 0.8.

### Study procedures

Initially, brochures were prepared based on the expertise of specialists and credible references. The content for the system was prepared similarly. Questionnaires were then distributed to parents in both paper and electronic forms, and after one month, reminders and links to a second questionnaire were sent for reassessment of their knowledge. To follow the participants to ensure receiving the materials, we chose an internet-based social network that was used by a high percentage of parents for their children’s educational needs. The app was just used for adjusting more contribution of the recruited parents in our study.

### Statistical analysis

Parental responses were recorded as multiple-choice and Likert scale answers. Data were analyzed using parametric t-tests and non-parametric Mann-Whitney U tests across the three groups. SPSS version 24 was used for all analyses, and the significance level was set at *p* < 0.05.

## Results

In this study, the participation rate was 55%, with 230 participants ensuring the predicted sample size of 70 individuals per group was met. The average age of mothers was 36.42 years. Based on the categorization of content for parents, topics such as the importance of milk teeth, nutrition, the harms of sugary substances, introduction to pediatric oral diseases, prevention methods, and dental care were identified and provided through the system or brochures.

In Group C, as the control group, the average score changed a little from 57.65 in the first round to 56.14 in the second, with no significant difference observed. In Group B, the average score increased from 55.16 to 59.65, and the average rating improved from 62.03 to 90.15, indicating a positive effect of the brochure (*P* < 0.001). In Group A, the average score increased from 53.74 to 57.99, and the average rating from 68.35 to 95.48, showing a significant difference (*P* < 0.001). Additionally, there was no strong correlation between the level of education and the scores obtained (*P* > 0.07).

34.5% of parents reported that their children experienced gum bleeding while brushing or flossing. 49.1% of students had at least one filled tooth, and 32.8% had at least two extracted teeth, reflecting concerning statistics for a populous country like Iran. 36.9% of the children were affected by ECC, and 51.4% had more than one decayed tooth (See Table 1).

**Table 1:** Comparison of each group before and after the educational intervention

Group	Test	(*N*) Before	(*N*) After	Mean Before/After	*P*-Value
A	Independent Samples t-Test	81	82	53.74 / 57.99	< 0.001
	Mann-Whitney U	81	82	68.35 / 95.48	< 0.001
B	Independent Samples t-Test	76	75	55.16 / 59.65	< 0.001
	Mann-Whitney U	76	75	62.03 / 90.15	< 0.001
C	Independent Samples t-Test	77	73	57.65 / 56.14	= 0.122
	Mann-Whitney U	77	73	79.95 / 70.81	= 0.197

Groups: A- Internet-Based Intervention; B- Brochure; C- Control (without any extra education)

## Discussion

### Objectives of the study

The practical aim of this paper was to examine the effectiveness of web-based education in enhancing parental awareness, and to determine whether such approaches can have a continuous and sustained impact at the community level. Researchers expect that the outcomes of these projects will integrate some of these educational methods into routine processes within the national education system. Potentially, similar methods may be pursued in the coming years for parent-specific education tailored to children at particular ages.

### Identifying educational needs

In this study, the educational needs of parents were identified based on the opinions of dental experts and a review of authoritative studies in the field. These needs included education on the importance of milk teeth, proper nutrition, the dangers of sugary substances, common oral diseases, preventive methods, and the importance of regular dental visits. Based on this categorization, appropriate educational content was designed and provided to parents through online systems and brochures.

### Results and their implications

The analysis of the results showed a significant increase in parental awareness following the intervention. This increase in knowledge was particularly evident in groups that received brochures and had access to the online system, consistent with the findings of Rothe et al. [33], Alqarni et al. [34], Abdul et al. [28] and Shirmohammadi et al. [14], demonstrating that short-term educational interventions can significantly enhance parental awareness. Furthermore, the study by Bagramian et al. [35] emphasizes that the high prevalence of dental decay among children can be attributed to a lack of parental awareness about the importance of oral health. The findings indicated that targeted education could effectively reduce these issues.

### Challenges in implementation

One of the critical issues that need attention is the practical difficulties in implementing web-based educational projects. These challenges include infrastructure provisioning, obtaining administrative approvals, securing support staff, ongoing website development, content management, and producing valuable content, which often requires more time and resources than initially estimated. It is essential to recognize that such projects should first be justified to managers and senior officials in supporting organizations and effectively implemented using specialized academic expertise and appropriate administrative communications. This ensures that they can be available as reliable educational resources.

Despite the efforts to implement these educational initiatives effectively, certain limitations must be acknowledged. These may include potential biases in the self-reported data from parents, variations in access to technology among different demographics, and the limited scope of the questionnaire used, which may not capture all relevant factors affecting parents’ awareness of oral health care.

### Technological advantages and policy implications

This study highlights the importance of utilizing modern technologies in delivering educational content. Online systems, due to their easier access, capability to offer more diverse content, and lower costs, have been recognized as more efficient tools compared to traditional paper brochures. This aligns with the results from studies by AlKlayb et al. [13], Calderon et al. [36], Underwood et al. [37] and which demonstrates that the use of digital media can significantly increase the effectiveness of educational efforts. It is hoped that with proper and forward-thinking policy-making, such projects will gain more attention and their results will be continuously accessible and usable. Therefore, increasing organizational support and funding can significantly impact the success of these initiatives.

## Conclusion

To ensure effective use of resources, parent education should be implemented in a strategic manner. Our study demonstrates that web-based systems and mobile applications are superior methods for enhancing parental awareness due to their higher accessibility and interactivity. These digital tools offer a more efficient and engaging approach compared to traditional methods, leading to significant improvements in parental knowledge about oral health care for children. Therefore, integrating such web-based educational methods into broader educational frameworks can maximize their impact and provide a cost-effective solution for increasing parental awareness.

## Data Availability

We prefer not to share our research data publicly; we are writing to declare that we have exact and clean research data available for probable assessment by the journal team.If the editorial team needs access to our data, please email the corresponding author for further evaluation.
